# Supplementary material for: In Vitro Antimelanoma Properties of Verbena officinalis Fractions
Source: Molecules. 2022 Sep 26;27(19):6329. doi: 10.3390/molecules27196329 (PMC9571856; doi:10.3390/molecules27196329)
Supplement: Supplementary file 1 [file molecules-27-06329-s001.zip › molecules-1900139-supplementary.pdf]

Antimelanoma properties of *Verbena officinalis* fractions

Rabia Nisar[1], Sanjay Adhikary[2], Saeed Ahmad[1], Mohammad Abrar Alam[2]

[1]Department of Pharmaceutical Chemistry, Faculty of Pharmacy, The Islamia University of Bahawalpur, Bahawalpur 63100, Pakistan

[2]Department of Chemistry and Physics, College of Sciences and Mathematics, Arkansas State University, Jonesboro, Arkansas, 72404, United States

ESI mass spectra of HA fraction of *V. officinalis*

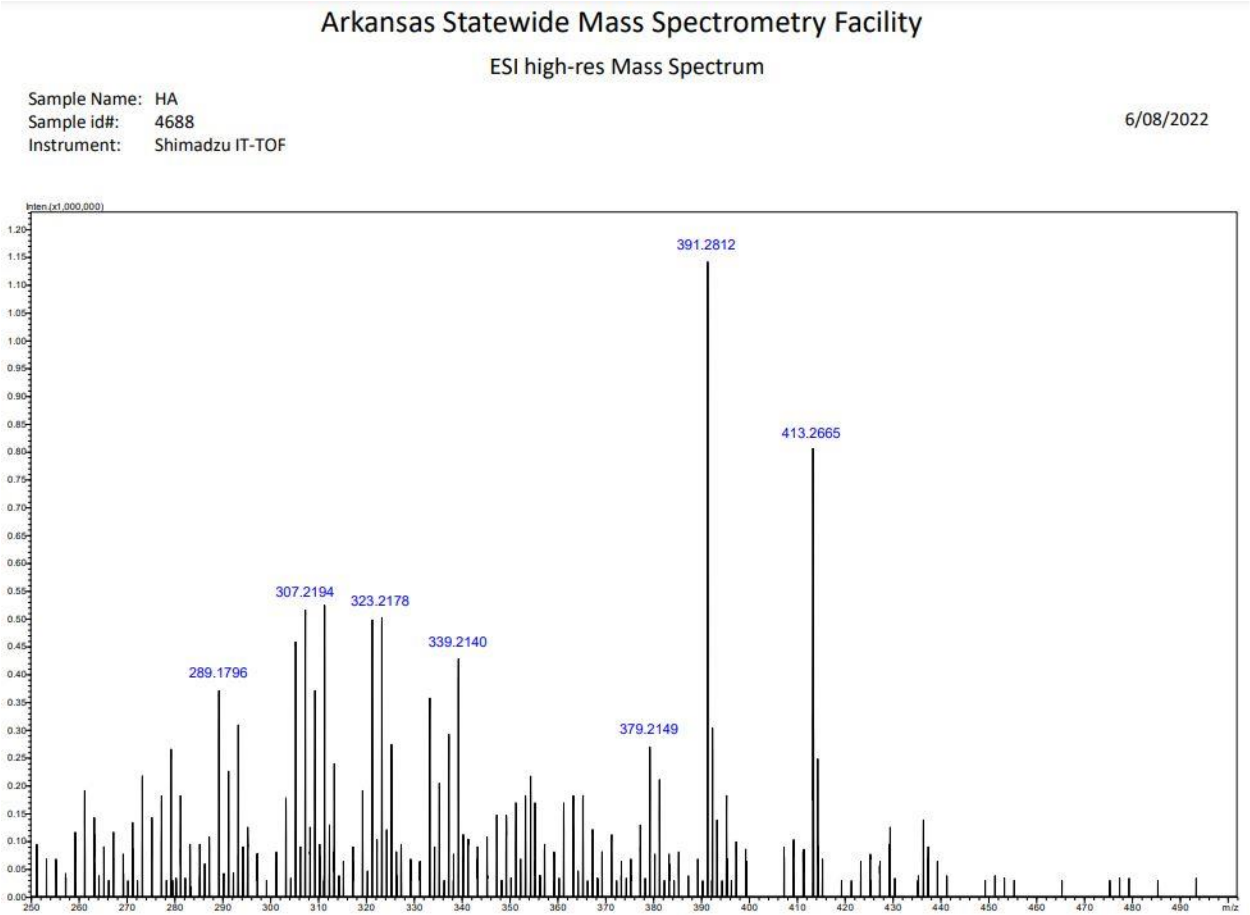

Figure S1. ESI high-resolution mass spectrum of HA extract of *V. officinalis*

1. **LC chromatogram and ESI mass spectra of VO79 isolated from chloroform extract of *V. officinalis* at different retention time**

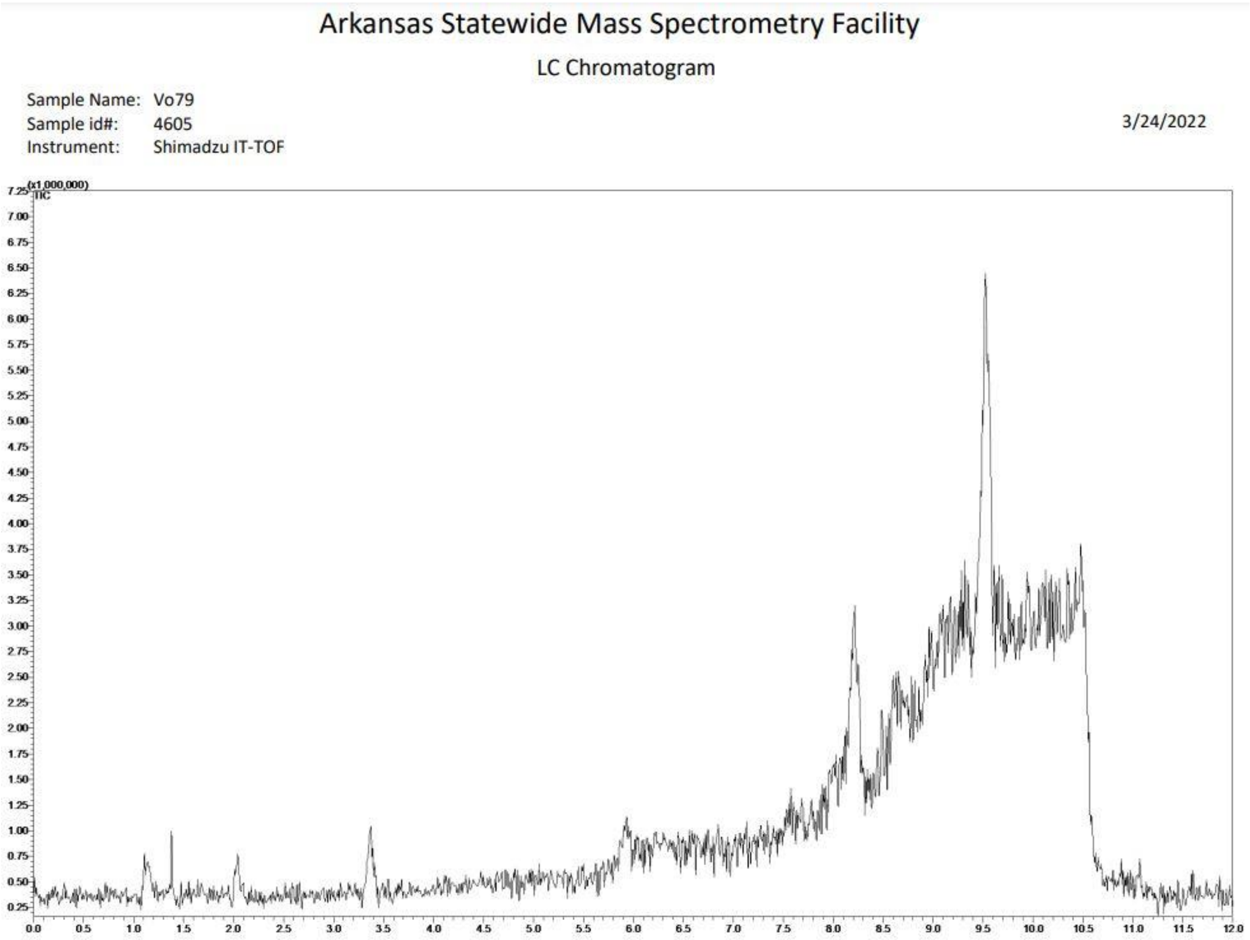

Figure S2. LC chromatogram of VO79 isolated from the chloroform extract of *V. officinalis*

Arkansas Statewide Mass Spectrometry Facility

ESI Mass Spectrum (3.37 min)

Sample Name: Vo79
Sample id#: 4605
Instrument: Shimadzu IT-TOF

3/24/2022

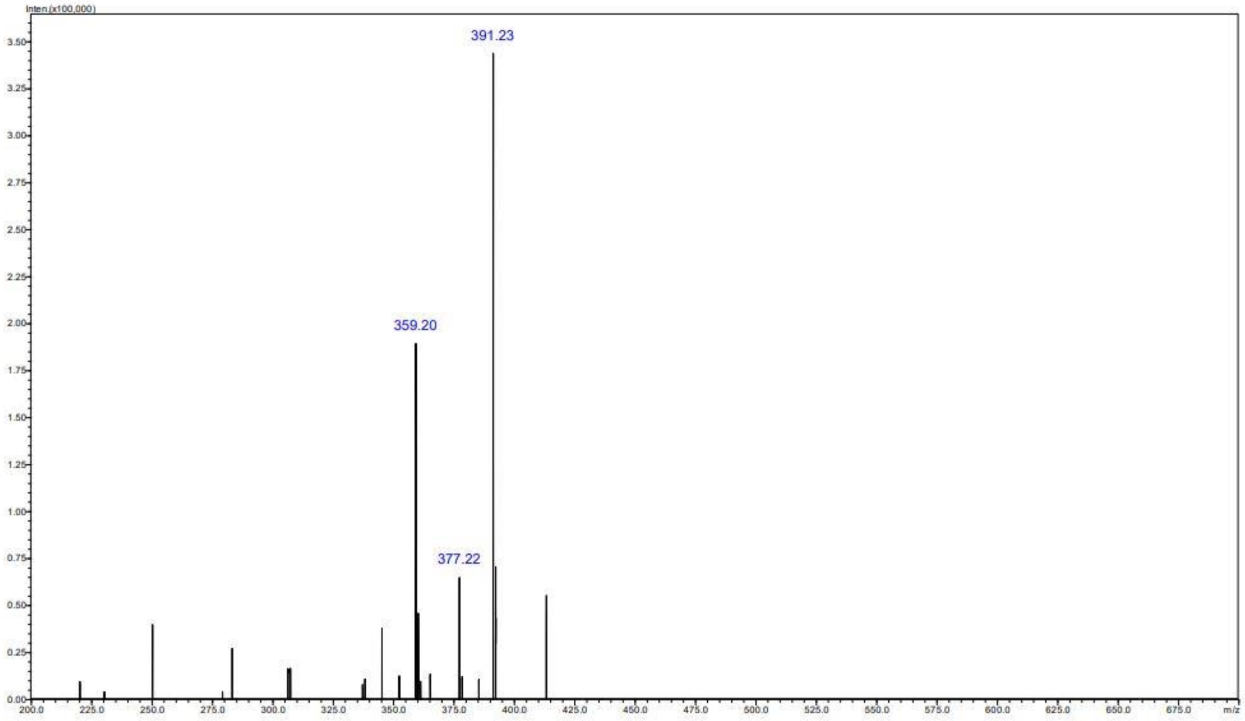

Figure S3. ESI mass spectrum of VO79 at retention time 3.37 min

Arkansas Statewide Mass Spectrometry Facility

ESI Mass Spectrum (8.21 min)

Sample Name: Vo79
Sample id#: 4605
Instrument: Shimadzu IT-TOF

3/24/2022

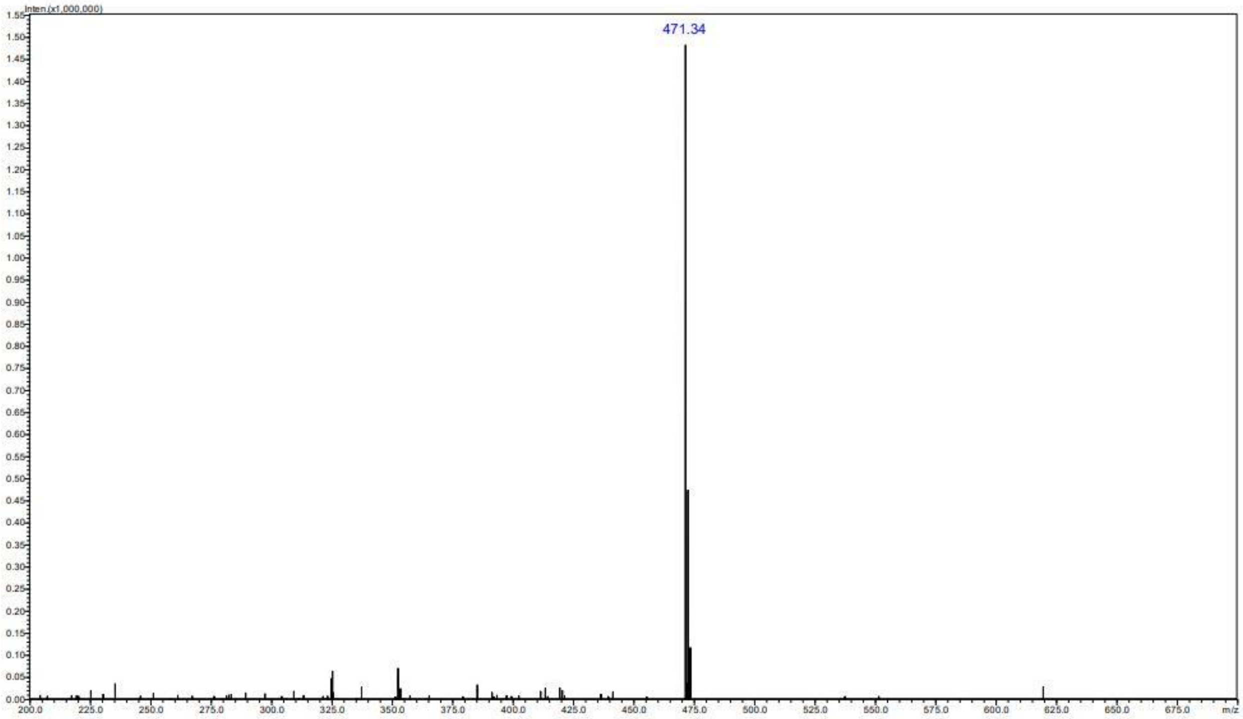

Figure S4. ESI mass spectrum of VO79 at retention time 8.21 min

Arkansas Statewide Mass Spectrometry Facility

ESI Mass Spectrum (9.53 min)

Sample Name: Vo79
Sample id#: 4605
Instrument: Shimadzu IT-TOF

3/24/2022

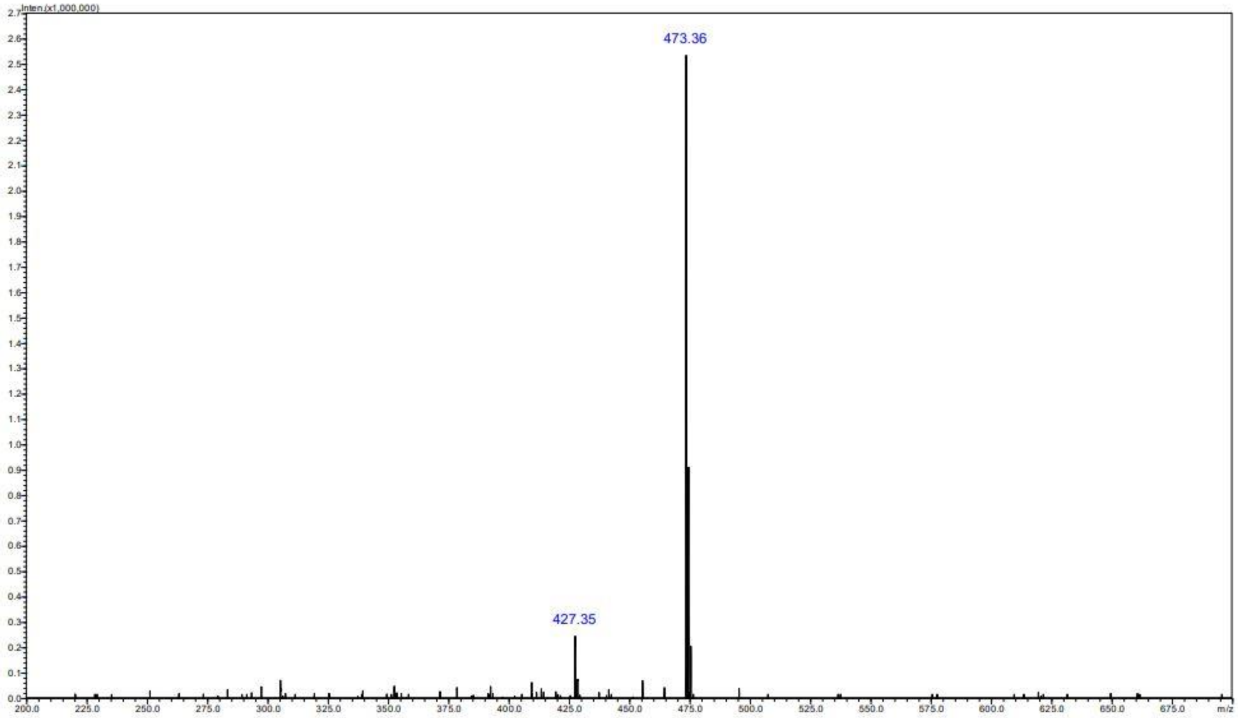

Figure S5. ESI mass spectrum of VO79 at retention time 9.53 min

2. LC chromatogram and ESI mass spectra of EA3 isolated from ethyl acetate extract of *V. officinalis* at different retention time

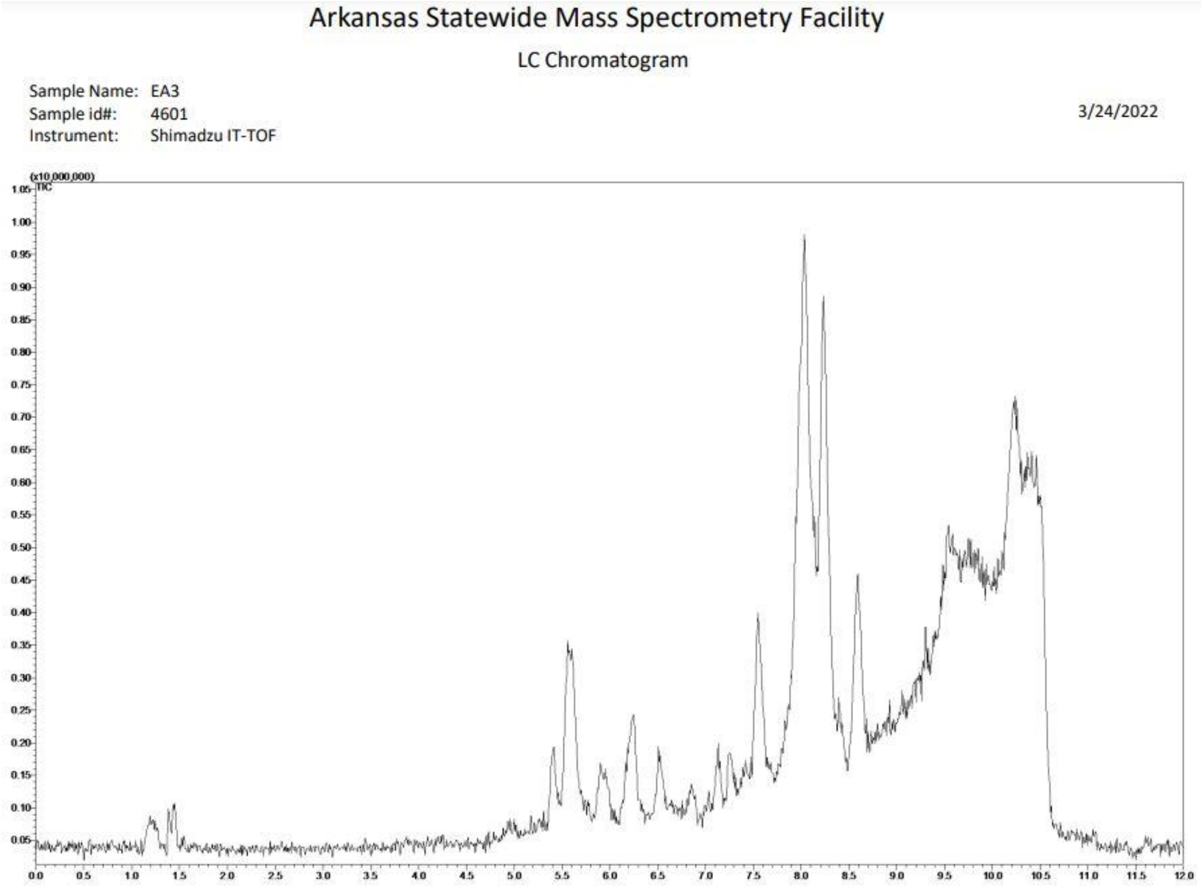

Figure S6. LC chromatogram of EA3 isolated from the ethyl acetate extract of *V. officinalis*

Arkansas Statewide Mass Spectrometry Facility

ESI Mass Spectrum (5.41 min)

Sample Name: EA3
Sample id#: 4601
Instrument: Shimadzu IT-TOF

3/24/2022

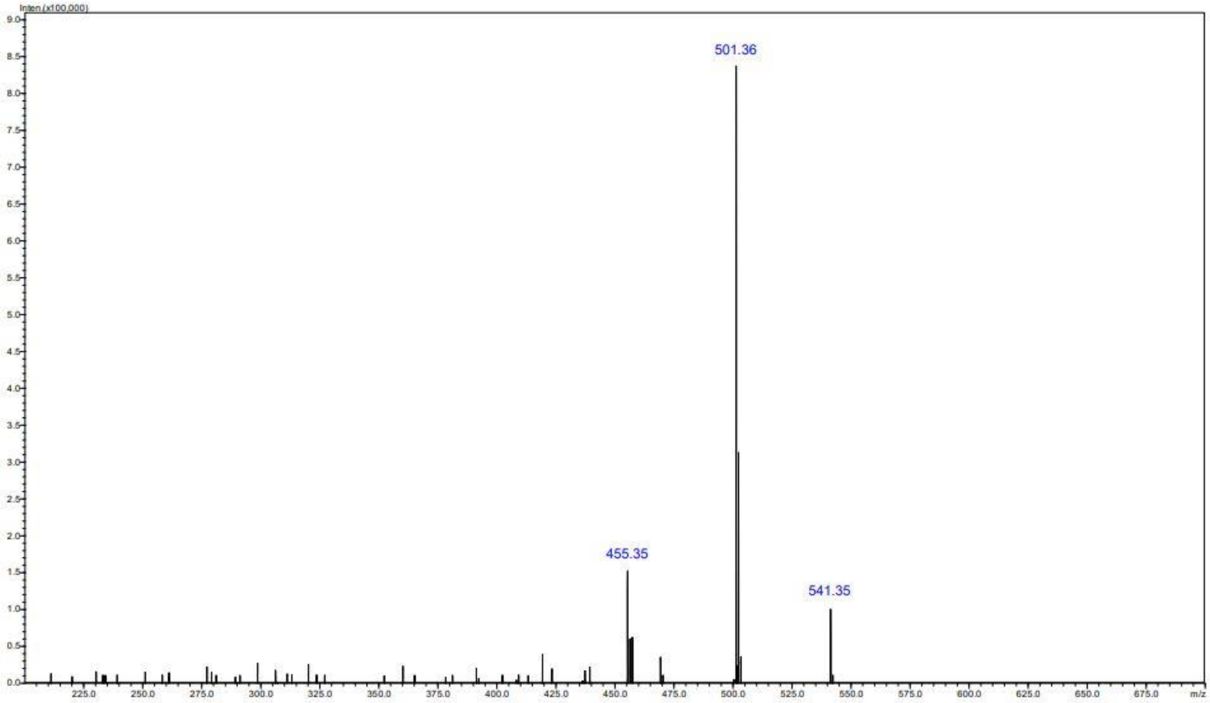

Figure S7. ESI mass spectrum of EA3 at retention time 5.41 min

Arkansas Statewide Mass Spectrometry Facility

ESI Mass Spectrum (5.58 min)

Sample Name: EA3
Sample id#: 4601
Instrument: Shimadzu IT-TOF

3/24/2022

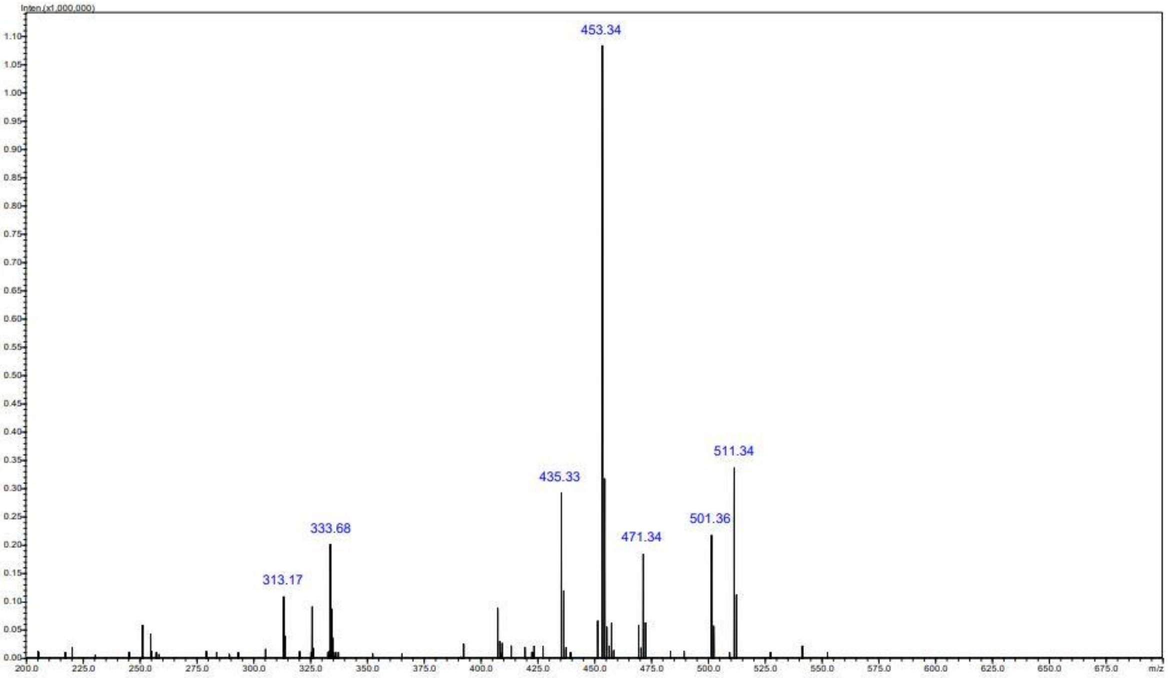

Figure S8. ESI mass spectrum of EA3 at retention time 5.58 min

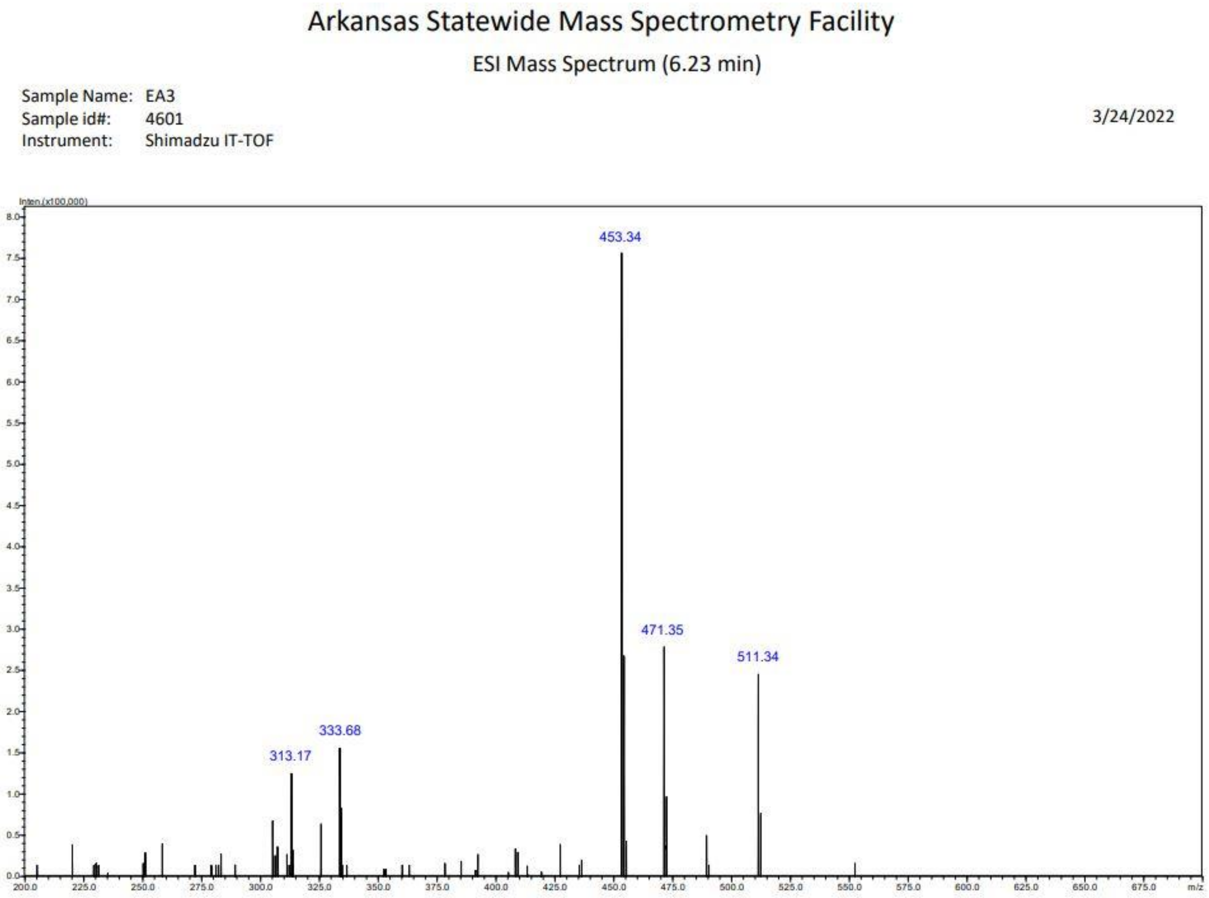

Figure S9. ESI mass spectrum of EA3 at retention time 6.23 min

Arkansas Statewide Mass Spectrometry Facility

ESI Mass Spectrum (6.52 min)

Sample Name: EA3
Sample id#: 4601
Instrument: Shimadzu IT-TOF

3/24/2022

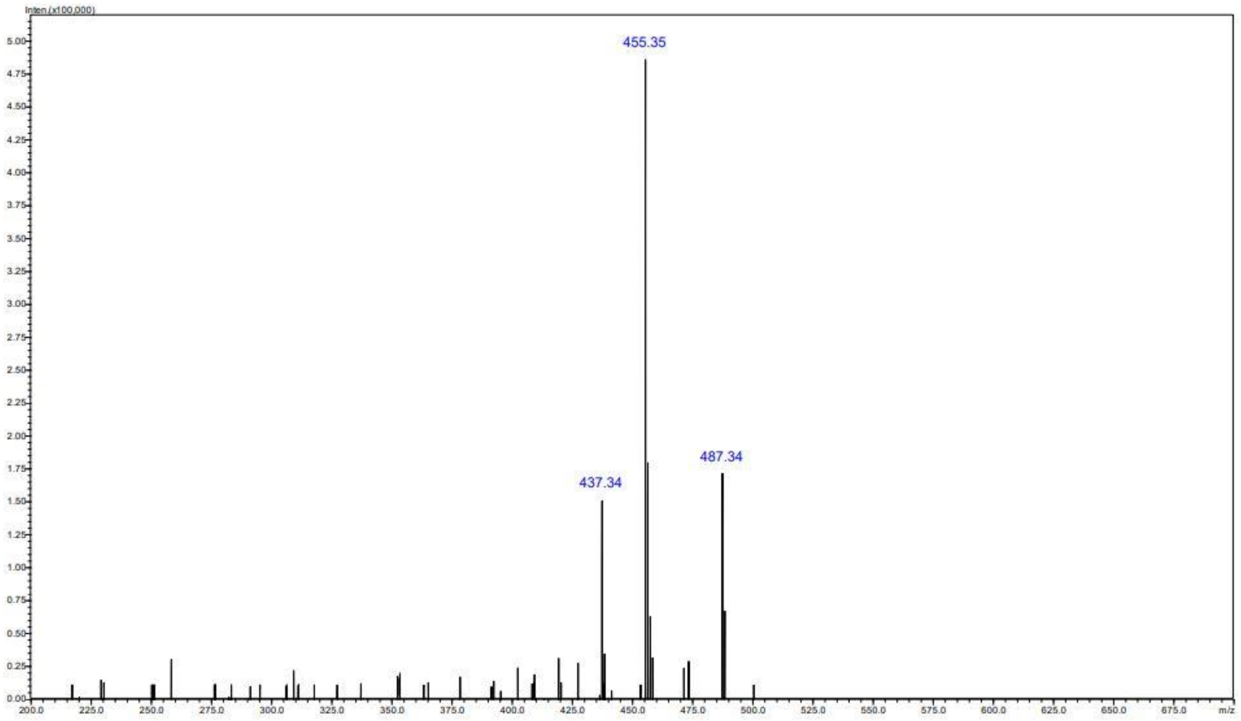

Figure S10. ESI mass spectrum of EA3 at retention time 6.52 min

Arkansas Statewide Mass Spectrometry Facility

ESI Mass Spectrum (6.86 min)

Sample Name: EA3
Sample id#: 4601
Instrument: Shimadzu IT-TOF

3/24/2022

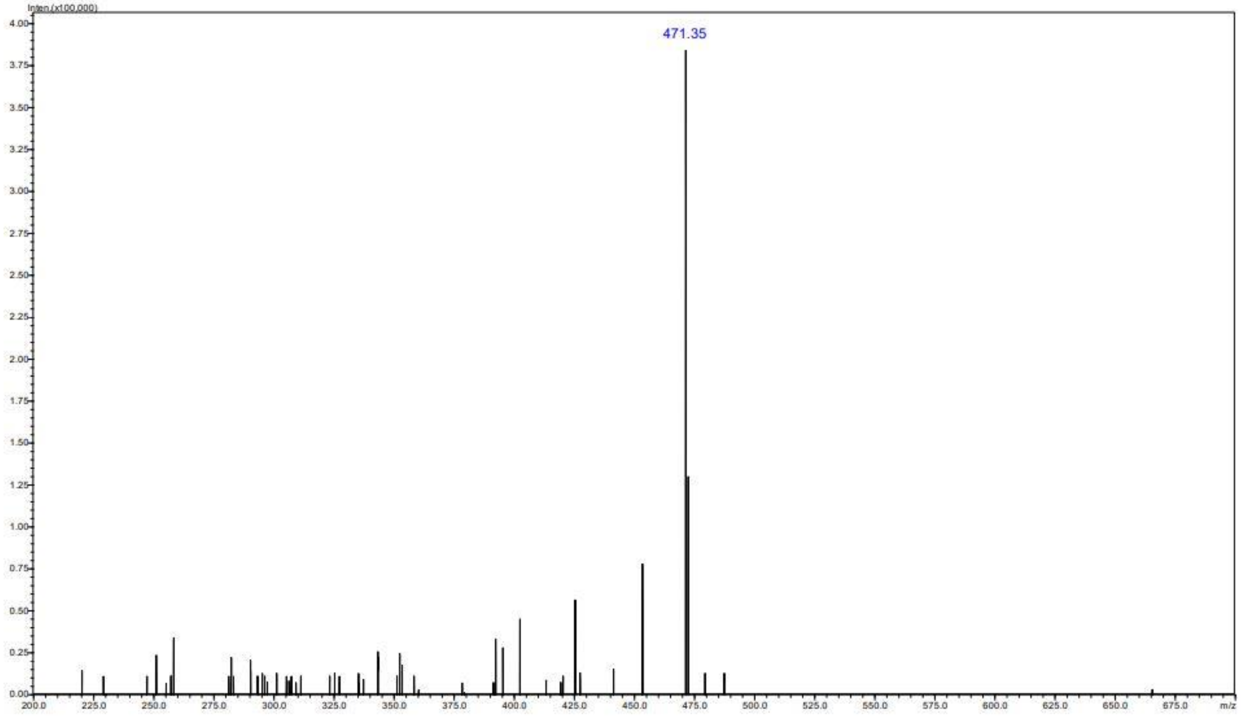

Figure S11. ESI mass spectrum of EA3 at retention time 6.86 min

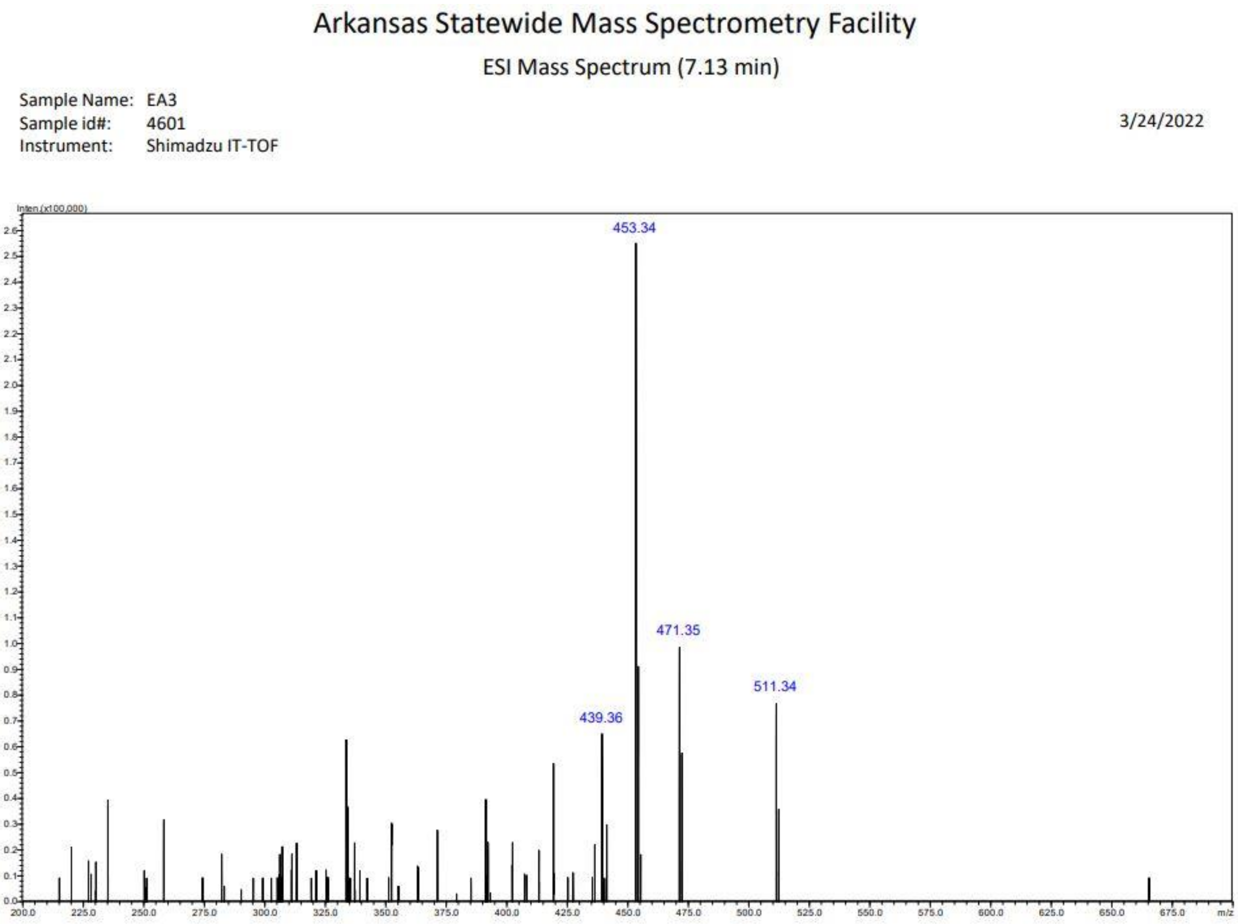

Figure S12. ESI mass spectrum of EA3 at retention time 7.13 min

Arkansas Statewide Mass Spectrometry Facility

ESI Mass Spectrum (7.55 min)

Sample Name: EA3
Sample id#: 4601
Instrument: Shimadzu IT-TOF

3/24/2022

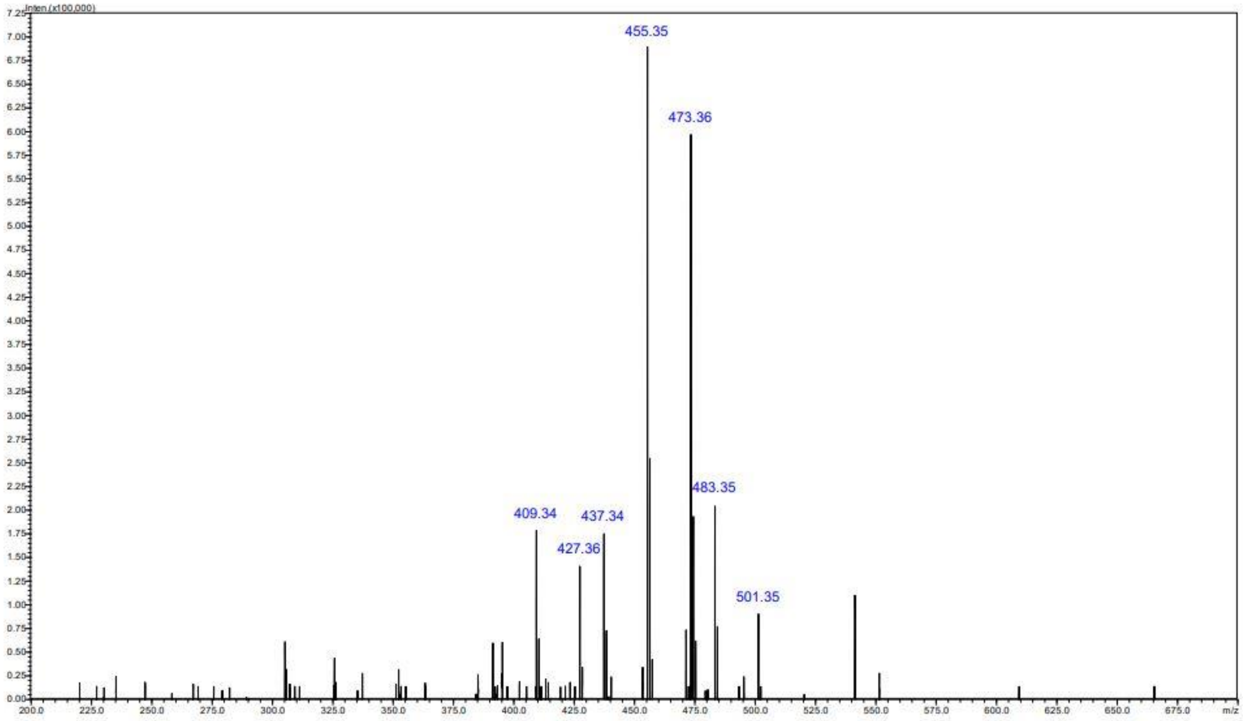

Figure S13. ESI mass spectrum of EA3 at retention time 7.55 min

Arkansas Statewide Mass Spectrometry Facility

ESI Mass Spectrum (7.55 min)

Sample Name: EA3
Sample id#: 4601
Instrument: Shimadzu IT-TOF

3/24/2022

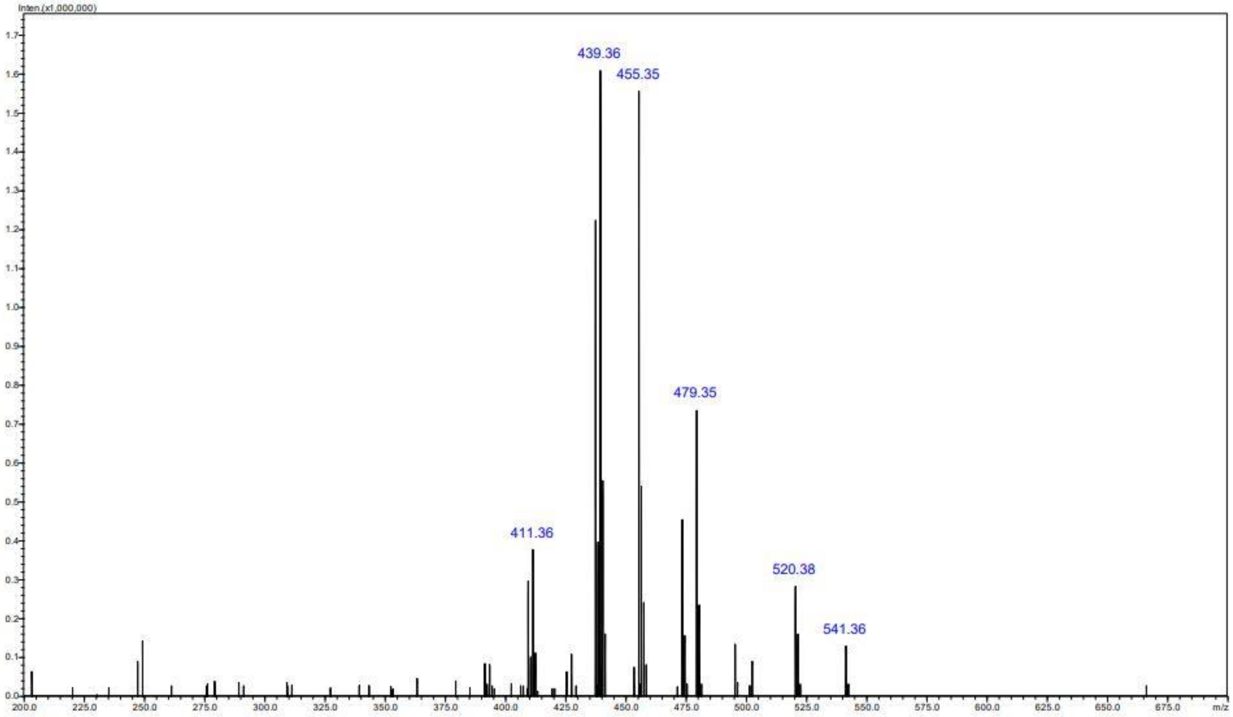

Figure S14. ESI mass spectrum of EA3 at retention time 7.55 min

Arkansas Statewide Mass Spectrometry Facility

ESI Mass Spectrum (8.23 min)

Sample Name: EA3
Sample id#: 4601
Instrument: Shimadzu IT-TOF

3/24/2022

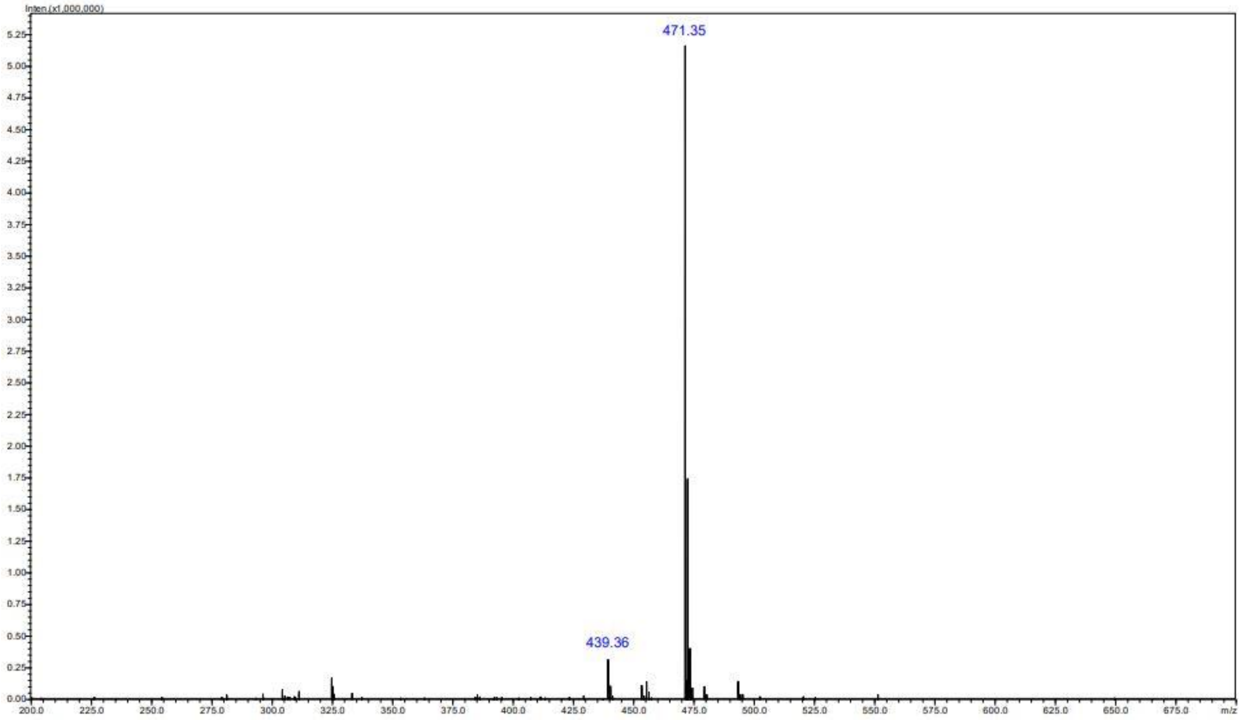

Figure S15. ESI mass spectrum of EA3 at retention time 8.23 min

Arkansas Statewide Mass Spectrometry Facility

ESI Mass Spectrum (8.59 min)

Sample Name: EA3
Sample id#: 4601
Instrument: Shimadzu IT-TOF

3/24/2022

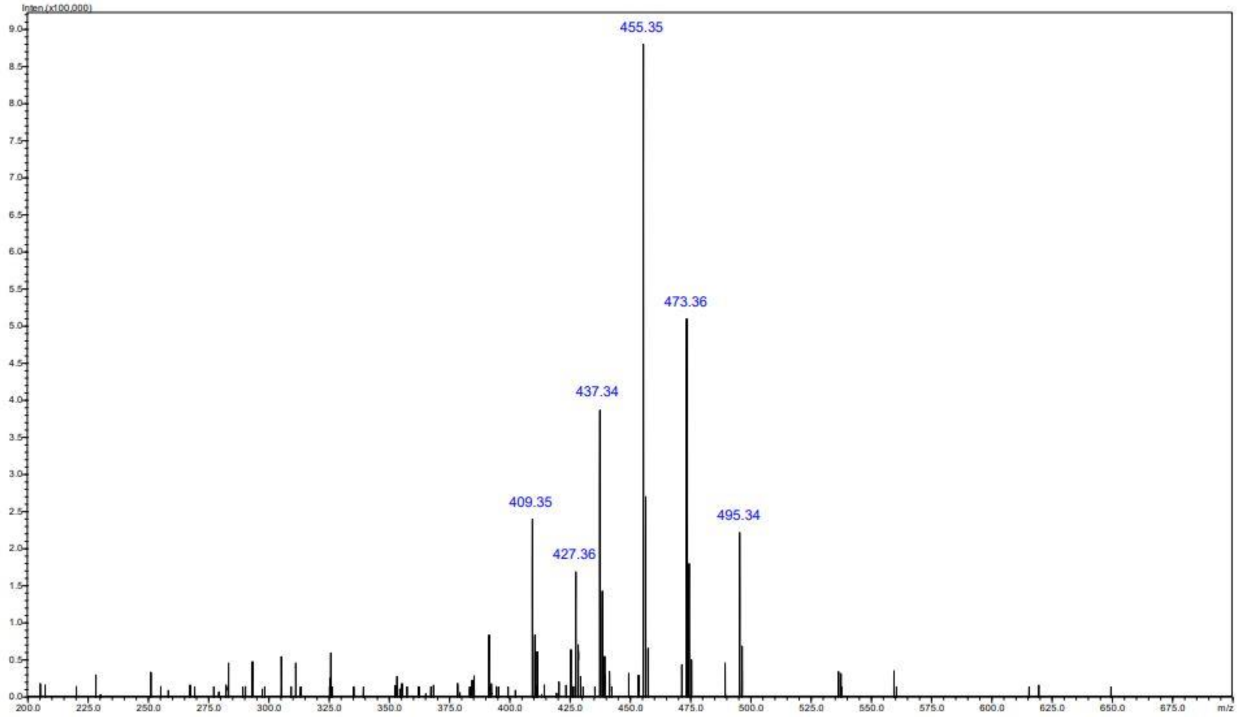

Figure S16. ESI mass spectrum of EA3 at retention time 8.59 min

Arkansas Statewide Mass Spectrometry Facility

ESI Mass Spectrum (10.23 min)

Sample Name: EA3
Sample id#: 4601
Instrument: Shimadzu IT-TOF

3/24/2022

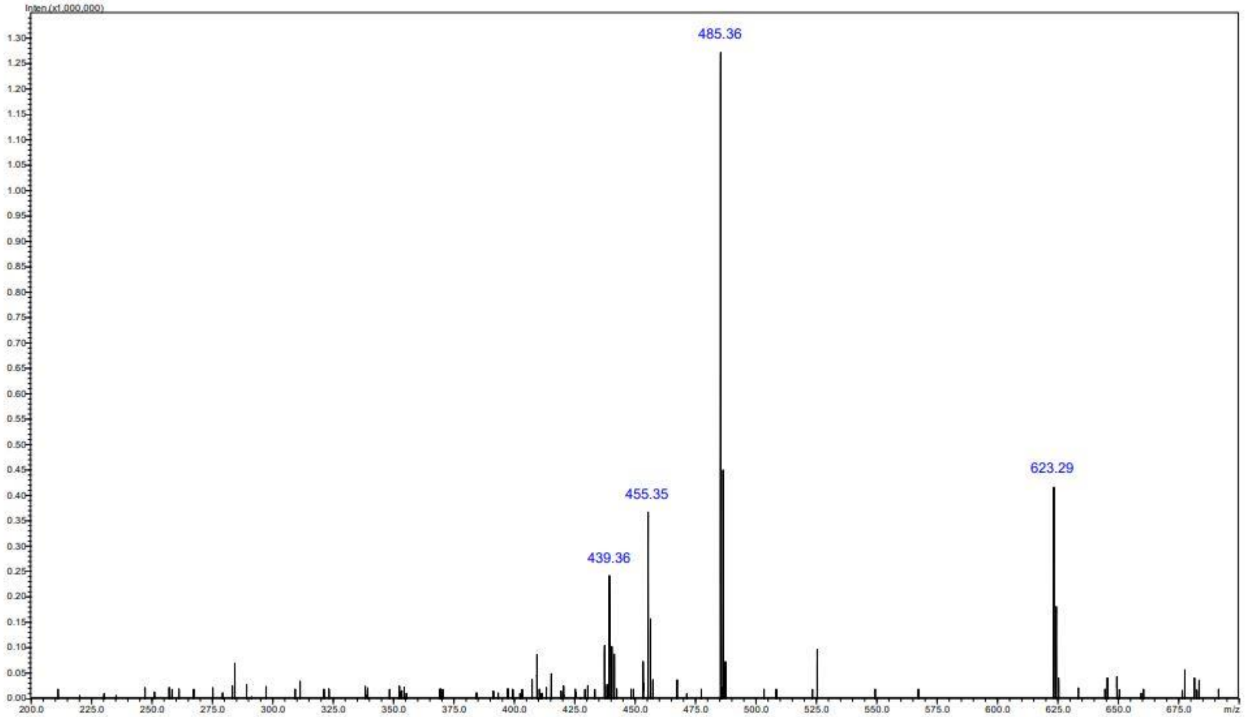

Figure S17. ESI mass spectrum of EA3 at retention time 10.23 min
